# Porous gold nanoparticles for attenuating infectivity of influenza A virus

**DOI:** 10.1186/s12951-020-00611-8

**Published:** 2020-03-24

**Authors:** Jinyoung Kim, Minjoo Yeom, Taeksu Lee, Hyun-Ouk Kim, Woonsung Na, Aram Kang, Jong-Woo Lim, Geunseon Park, Chaewon Park, Daesub Song, Seungjoo Haam

**Affiliations:** 1 Department of Chemical and Biomolecular Engineering, Yonsei University, Yonsei-ro 50, Seoul, 120-749 Republic of Korea; 2 Department of Pharmacy, Korea University College of Pharmacy, Sejong-ro 2511, Sejong, 30019 Republic of Korea; 3 Department of Nano Manufacturing Technology, Korea Institute of Machinery and Materials (KIMM), 156 Gajeongbuk-Ro, Yuseong-Gu, Daejeon, 34103 Republic of Korea; 4 College of Veterinary Medicine, Chonnam National University, Yongbong-ro 77, Buk-Gu, Gwangju, South Korea

**Keywords:** Virus inactivation, Influenza A virus, Porous gold nanoparticle, Membrane fusion, Disulfide bond

## Abstract

**Background:**

Influenza viruses (IVs) have become increasingly resistant to antiviral drugs that target neuraminidase and matrix protein 2 due to gene mutations that alter their drug-binding target protein regions. Consequently, almost all recent IV pandemics have exhibited resistance to commercial antiviral vaccines. To overcome this challenge, an antiviral target is needed that is effective regardless of genetic mutations.

**Main body:**

In particular, hemagglutinin (HA), a highly conserved surface protein across many IV strains, could be an effective antiviral target as it mediates binding of IVs with host cell receptors, which is crucial for membrane fusion. HA has 6 disulfide bonds that can easily bind with the surfaces of gold nanoparticles. Herein, we fabricated porous gold nanoparticles (PoGNPs) via a surfactant-free emulsion method that exhibited strong affinity for disulfide bonds due to gold–thiol interactions, and provided extensive surface area for these interactions. A remarkable decrease in viral infectivity was demonstrated by increased cell viability results after exposing MDCK cells to various IV strains (H1N1, H3N2, and H9N2) treated with PoGNP. Most of all, the viability of MDCK cells infected with all IV strains increased to 96.8% after PoGNP treatment of the viruses compared to 33.9% cell viability with non-treated viruses. Intracellular viral RNA quantification by real-time RT-PCR also confirmed that PoGNP successfully inhibited viral membrane fusion by blocking the viral entry process through conformational deformation of HA.

**Conclusion:**

We believe that the technique described herein can be further developed for PoGNP-utilized antiviral protection as well as metal nanoparticle-based therapy to treat viral infection. Additionally, facile detection of IAV can be achieved by developing PoGNP as a multiplatform for detection of the virus.

## Introduction

Acute respiratory diseases comprise over 75% of overall infectious disease occurrences in developed countries, of which 80% are caused by viruses [1]. Above all, the influenza virus (IV) is recognized as a serious public health concern with 200,000 hospitalizations and 36,000 deaths annually in the United States alone [2]. To date, conventional commercialized antiviral agents for IV have been developed to control IV pandemics by blocking two different targets: matrix protein 2 (M2) ion channel and neuraminidase (NA) [3]. Transportation of H^+^ ions across the transmembrane is blocked by the M2 ion channel inhibitor, which results in incomplete viral uncoating and prevents viral replication. The NA inhibitor interrupts the hydrolysis of terminal sialic acid residues on new IV, thus preventing the release of viruses from the infected host cell. Promotion of IV infection is interrupted with these inhibitors. However, the U.S. Centers for Disease Control (CDC) announced that over 99% of IV strains H3N2 and H1N1 present resistance to M2 ion channel inhibitors, including amantadine and rimantadine [4]. Furthermore, recent influenza strains, including those responsible for the pandemic outbreaks in 2009, showed enormously high resistance to the commercialized NA inhibitor, Tamiflu (oseltamivir) [5, 6]. Mutation of viral surface proteins has triggered incremental IV resistance to conventional drug treatments by transforming the drug binding site in its protein, hence increasing viral infectivity and mortality [7–9].

Hemagglutinin (HA), one of the viral surface proteins deeply involved in membrane fusion with the host cell, has been investigated as another potentially effective antiviral strategy. During the fusion process, disulfide bonds linking the two different parts of HA cleave to unfold the HA molecule and the fusion peptide hidden within causes membrane fusion to spread viral RNA to the host cell [10–12]. Disulfide bonds have a crucial role in the membrane fusion process as previously described [13–15], thus we focused on HA disulfide bonds as an antiviral target herein.

Meanwhile, inorganic metals such as gold and silver have been investigated for their ability to neutralize HA activity by cleaving HA disulfide bonds using their strong affinity for gold and silver. Especially, it has been reported that the silver-thiol interaction between HA disulfide bonds and silver nanoparticles (AgNPs) could inhibit IV infection by cleaving disulfide bonds [16–18]. Therefore, various modifications of noble metallic nanoparticles have been tested to mitigate viral infection, i.e., tannic acid-modified silver nanoparticle [19], silver nanoparticle/chitosan composite [20], curcumin-modified silver nanoparticle [21], PVP-coated silver nanoparticle [22, 23] multivalent gold nanoparticles [24–27] and gallic acid-functionalized gold nanoparticle [28]. These noble metal-based nanoparticles suppressed viral infection by blocking viral entry to the host cell.

Here, for an improved antiviral treatment agent using metallic nanoparticles, we introduced the porous gold nanoparticle (PoGNP) for cleaving disulfide bonds. PoGNPs permit a facile fabrication process that does not require additional heat or surfactant, while Ag-based nanoparticles should be modified with a capping agent for stabilization under in vitro conditions and to reduce their cytotoxicity. In addition, PoGNPs possess extensive surface area due to their unique nanobundled structure [29, 30]. Moreover, PoGNPs are expected to have high affinity for disulfide bonds due to gold-disulfide bond formation and their extensive surface area can effectively inactivate the IV by cleaving disulfide bonds, which blocks membrane fusion and viral entry to the host cell.

**Scheme 1:**
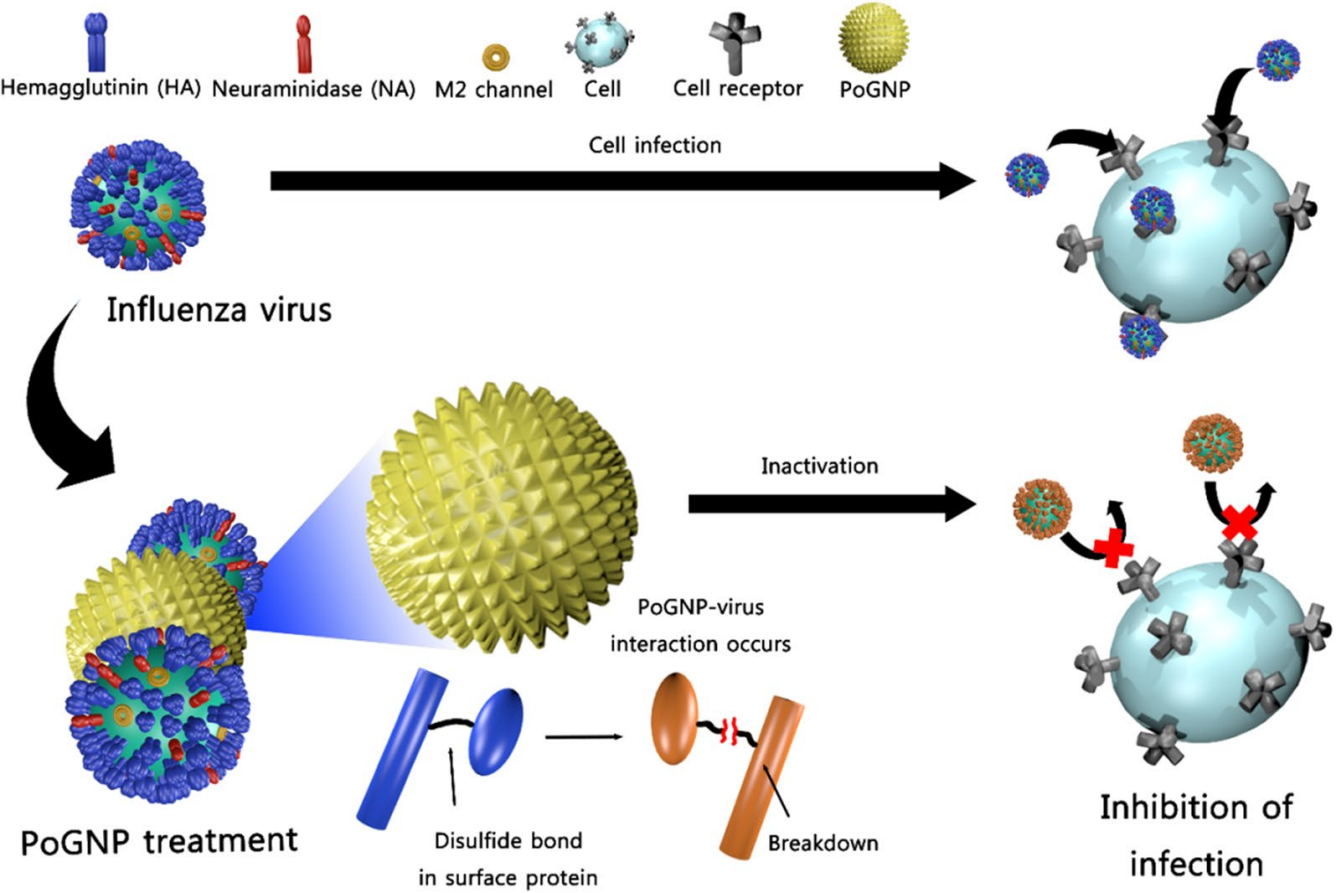
Schematic illustration of inactivation of influenza A virus (IAV) treated with porous gold nanoparticle (PoGNP). PoGNP interacts with IAV surface proteins and cleaves their disulfide bonds. Inactivated viruses exhibit lower infectivity to cells

In the present study, influenza A virus (IAV) was treated with PoGNP to show that PoGNP inactivates the virus (Scheme 1). To visualize this inactivation ability, the amount of post-infection intracellular viral RNA was measured after nanoparticle treatment. In addition, various IV strains, including H1N1, H3N2, and H9N2, were analyzed to confirm the inhibition efficacy of PoGNP regardless of IAV mutation.

## Results and discussion

### Preparation and characterization of porous gold nanoparticle (PoGNP) and other metallic nanoparticles

In contrast to conventional gold nanoparticle synthesis, PoGNP was synthesized using the surfactant-free emulsion method. The Au^3+^ ion in HAuCl_4_ and anilinium ion (C_6_H_5_NH_3_^+^) formed the PANI-Au complex nanoparticle by reducing the Au^3+^ ion and oxidizing the anilinium ion to PANI through redox reaction [29]. After fabrication of the PANI-Au complex nanoparticle, PANI was selectively etched by NMP from the PANI-Au nanocomplex, shaping a mesoporous gold structure. Non-porous sGNP was synthesized via the seed-mediated growth method to compare whether nanoparticle morphology could affect inactivation of the virus. AgNP was also prepared as an antiviral control group as AgNP has been widely studied for its antiviral properties.

**Fig. 1:**
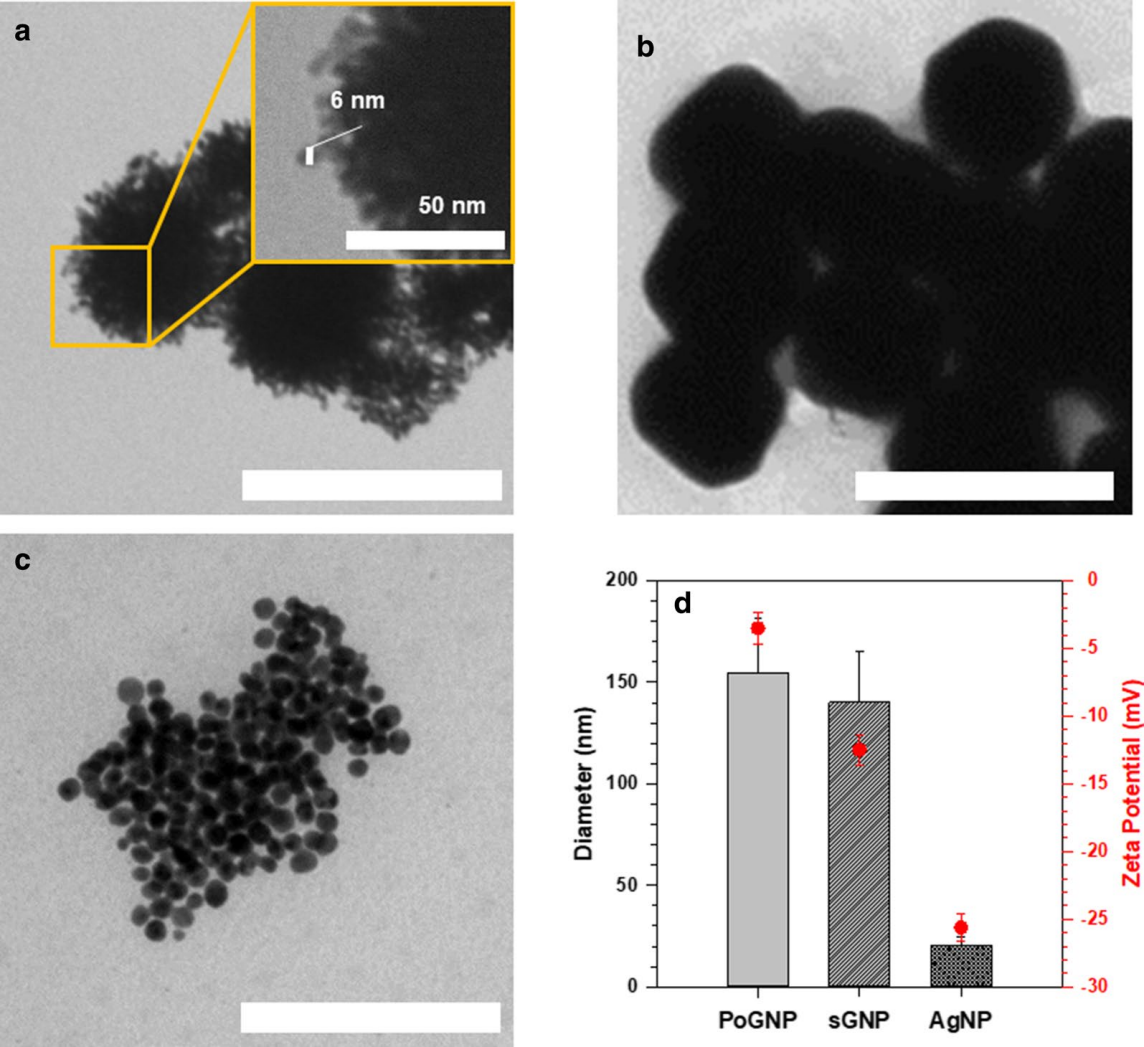
**a** TEM image of fabricated PoGNP. Inlet image shows nanobundles on the surface of PoGNP. **b** sGNP, **c** AgNP, **d** Mean of cumulative size of nanoparticles determined by DLS analysis. Zeta potential of each nanoparticle was also obtained. The unlabeled scale bars represent 200 nm. PoGNP, porous gold nanoparticle; sGNP, spherical gold nanoparticle; AgNP, silver nanoparticle

The morphology of the synthesized nanoparticles was observed by TEM and their average size was determined by dynamic light scattering (DLS) analysis. TEM images of synthesized PoGNP exhibited rough surfaces with 6 nm-sized nanosphere bundles, composing 150 nm sized spherical structure (Fig. 1a) whereas sGNP and AgNP exhibited narrow monodispersed spherical structures with smooth surfaces (Fig. 1b, c, Additional file 1: Figure S1). Cumulative size of PoGNP, AgNP, and sGNP was 154.24 ± 37.05 nm, 140.23 ± 25.10 nm and 20.52 ± 4.49 nm, respectively (Fig. 1d). Zeta potential of the formulated PoGNP was rather neutral charge (− 3.5133 ± 1.1548 mV), compared to sGNP (− 12.4733 ± 1.1243 mV) and AgNP (− 25.6167 ± 0.9810 mV).

Moreover, the biocompatibility of the synthesized nanoparticles was evaluated by MDCK cell viability assay. MDCK cells in 96-well plates were exposed to various nanoparticle concentrations and cell viability was measured by the WST-1 assay, with the optical density being measured at 450 nm. The measured cell viability after nanoparticle treatment was greater than 95%, except for 0.2 mg/mL PoGNP and 0.1–0.2 mg/mL AgNP, which were observed to have 80% cell viability. The results of the nanoparticle cell viability assay implied that the infectivity of nanoparticle-treated viruses could be evaluated by MDCK cytotoxicity assay because the nanoparticles exhibited good biocompatibility with the MDCK cells (Additional file 1: Figure S2).

### Interaction between synthesized nanoparticle and influenza virus

**Fig. 2:**
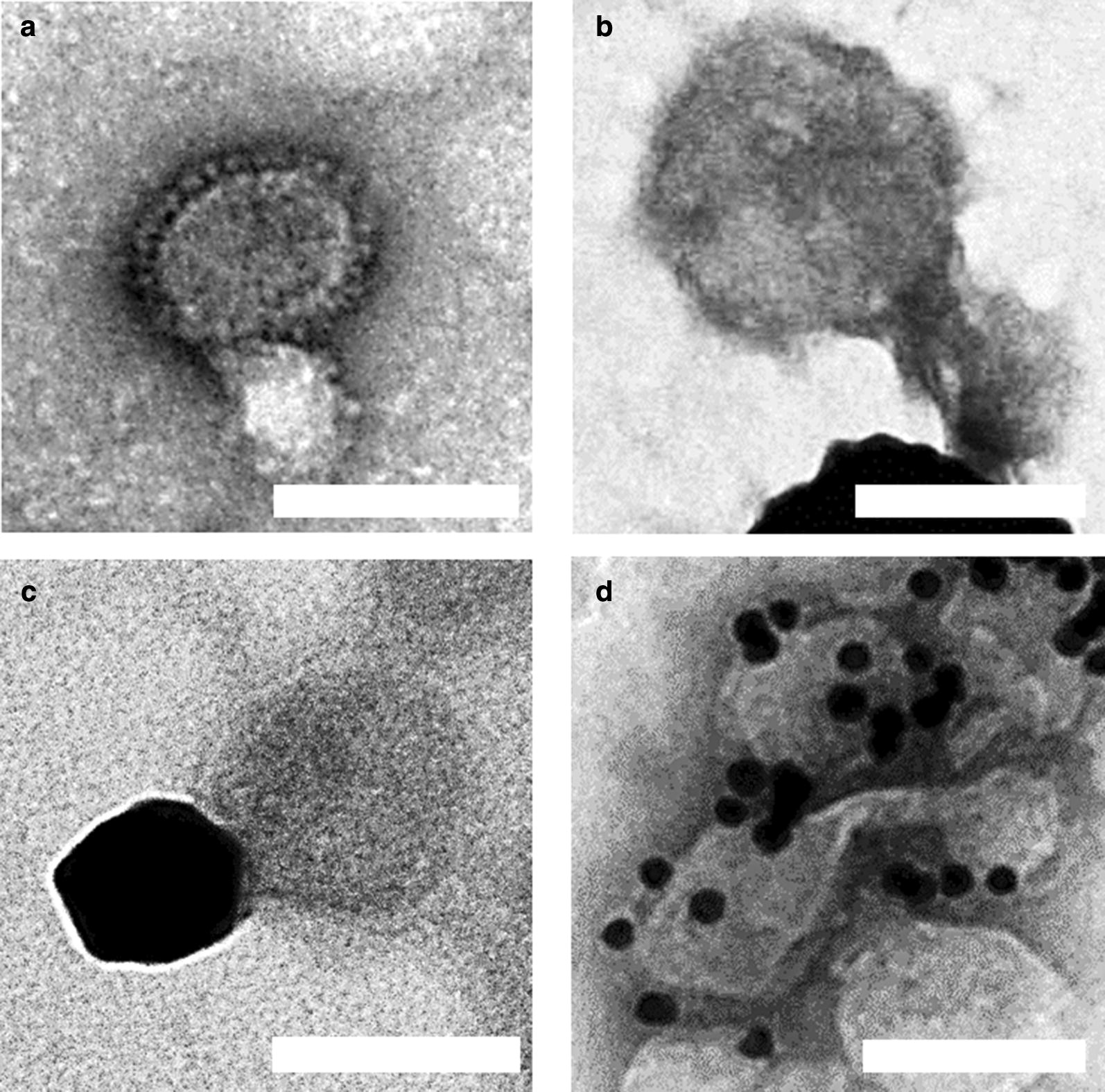
**a** TEM image of IAV. **b** PoGNP-treated IAV. **c** sGNP-treated IAV. **d** AgNP-treated IAV. All scale bars represent 100 nm. PoGNP, porous gold nanoparticle; *sGNP* spherical gold nanoparticle, *AgNP* silver nanoparticle

**Fig. 3:**
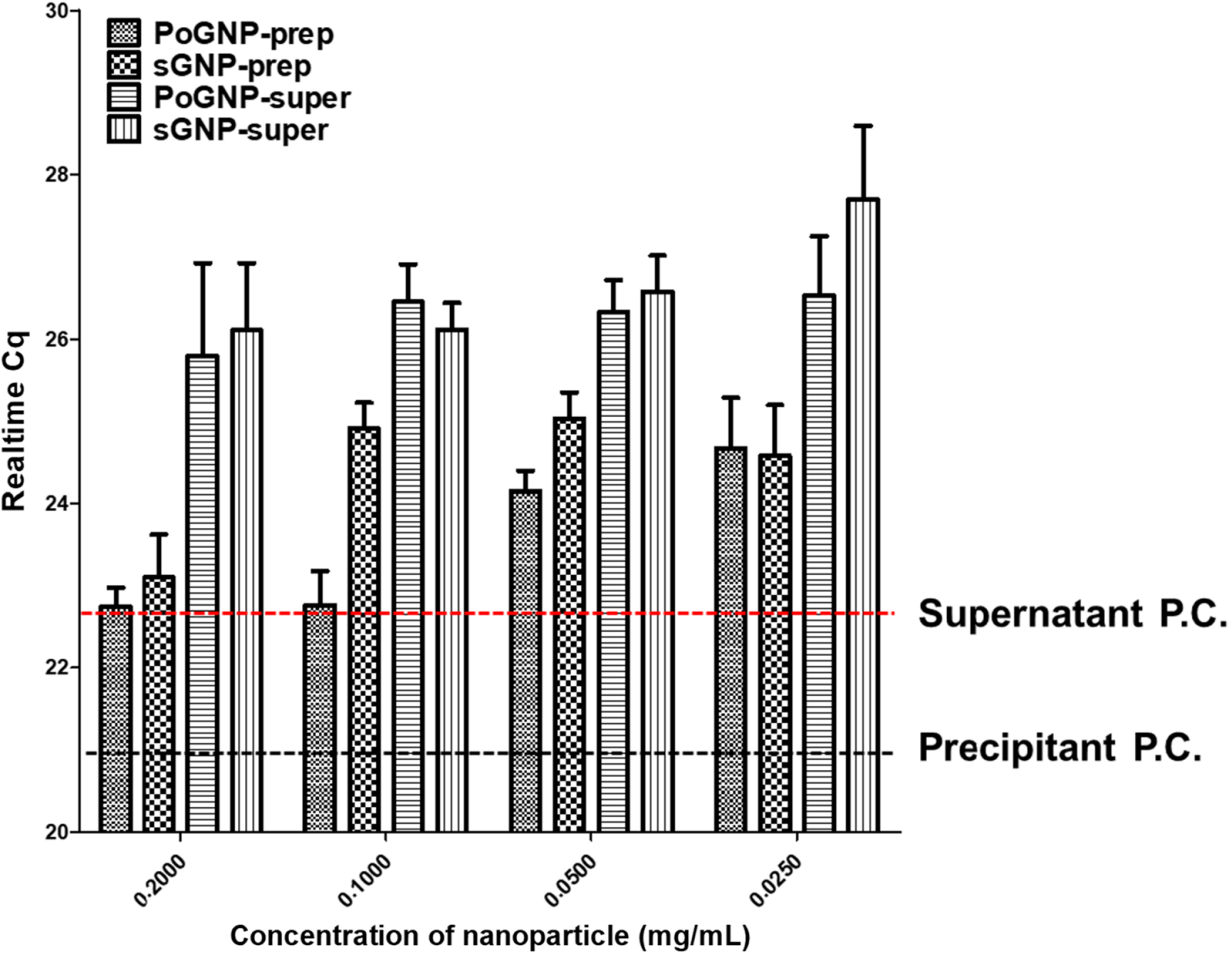
Real-time RT-PCR data of nanoparticle-treated IAVs after centrifugation. (Red dotted line: supernatant positive control, black dotted line: precipitate positive control, viral titer: 10^6^ EID_50_/mL). PoGNP-Prep, precipitate of PoGNP-treated IAV after centrifugation; sGNP-Prep, precipitate of sNGP-treated IAV after centrifugation; PoGNP-Super, supernatant of PoNGP-treated IAV after centrifugation; sGNP-Super, supernatant of sNGP-treated IAV after centrifugation. *PoGNP* porous gold nanoparticle, *sGNP* spherical gold nanoparticle, *AgNP* silver nanoparticle

Interaction between the prepared nanoparticles and IAV HA was evaluated, as we hypothesized inactivation of IAV to be induced by cleaving disulfide bonds required for viral membrane fusion with the host cell. TEM analysis was first conducted for a simple confirmation of the interaction between the virus and the nanoparticles. IAV suspension was incubated with 2 × 10^−1^ mg/mL of nanoparticles for 10 min, and then the mixtures were prepared for TEM analysis. However, interaction was difficult to observe in the TEM images with larger NPs such as PoGNP and sGNP. On the other hand, AgNP agglomerated on the viral surface as expected, since the small size of AgNP facilitated migration (Fig. 2). Another technique was considered to prove the affinity between HA and the larger sGNPs. As the isolation of IAV requires ultracentrifugation over 20,000*g* [31] or gradient centrifugation [32], we centrifuged the samples at 6000*g* for 10 min to monitor whether H3N2 precipitated with the nanoparticles. We assumed that the nanoparticle-treated H3N2 virus would precipitate with the nanoparticles in contrast to the H3N2 virus sample alone. Definitively, the real-time cycle quantification (C_q_) value of PoGNP-treated H3N2 virus in redispersed precipitate solution was much lower than that in the supernatant, indicating that H3N2 virus could interact with PoGNP (Fig. 3). In addition, PoGNP attracted more H3N2 virus than sGNP at lower concentration according to the real-time C_q_ values of precipitated samples, which indicated that PoGNP had much higher affinity for HA compared with sGNP. The difference in attraction resulted from their surface structure; the foam-shaped porous outer surface of PoGNP created more surface area for interaction with HA than the sGNP surface.

### Antiviral effect of PoGNP compared with other metallic nanoparticles

**Fig. 4:**
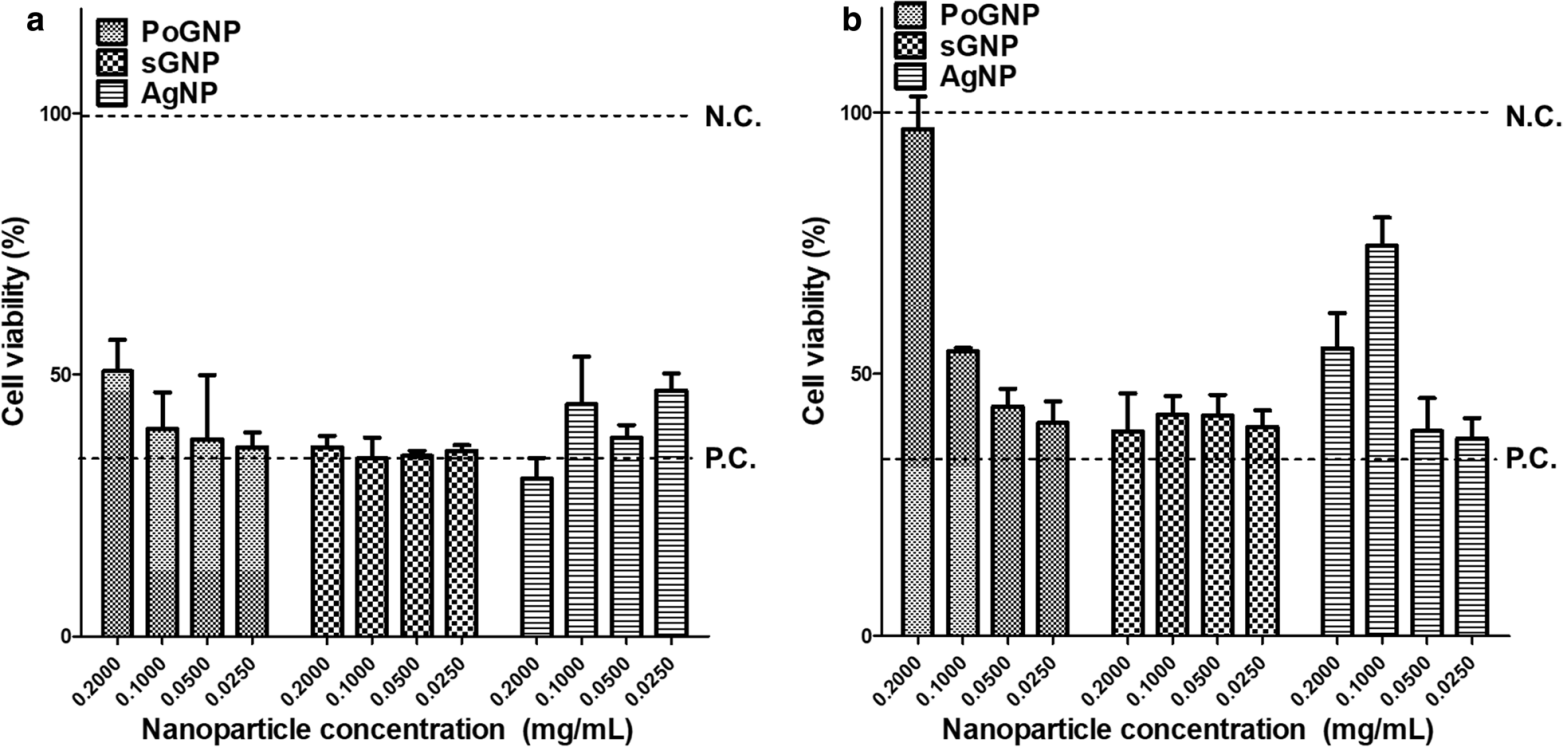
MDCK Cell viability assay of nanoparticle-treated H1N1 virus (viral titer: 10^6^ EID_50_/mL) for **a** 10 min and **b** 60 min. Upper line: negative control (N.C.), lower line: positive control (P.C.). *PoGNP* porous gold nanoparticle, *sGNP* spherical gold nanoparticle, *AgNP* silver nanoparticle

To observe the antiviral effect of nanoparticles, H1N1 virus was exposed to each nanoparticle suspension for 10 min and 60 min prior to infection of the MDCK cells. The antiviral effect of the nanoparticles was determined by WST-1 cytotoxicity assay by observing the optical density of treated cells at 450 nm. Compared with the other nanoparticles, PoGNP showed much higher antiviral activity on H1N1 virus, whereas AgNP showed only minor antiviral activity over 0.1 mg/mL AgNP. 0.2 mg/mL PoGNP successfully inactivated H1N1 virus after exposure for 60 min. In contrast, sGNP had no antiviral effect regardless of its concentration or exposure time (Fig. 4). Comparing PoGNP with sGNP, the difference in nanoparticle antiviral activity is the result of differences in their specific surface areas despite similar diameters; each nanoframe of PoGNP behaved as a single reactant for disulfide bonds that could interact with HA. AgNP was able to agglomerate on viral HA and had extensive specific surface area for interaction compared with sGNP due to its small size; however, PoGNP showed higher inactivation of the virus at 0.2 mg/mL. PoGNP’s superior virus inactivation ability compared with AgNP and sGNP is due to both its stability under saline conditions and its high affinity for HA. The antiviral effectiveness of AgNP is restricted because AgNP aggregated in the culture media at higher concentration and it should only be treated at concentrations lower than 0.1 mg/mL due to toxicity concerns. As observed by lower attraction of sGNP to viral HA in the binding efficiency test with centrifugation, sGNP exhibited lower antiviral activity, as expected. Indeed, the inactivation of H1N1 virus by sGNP was much lower than that of PoGNP, although sGNP also exhibited affinity for HA at higher concentration. This result endorsed the fact that inactivation of H1N1 virus was caused by the cleavage of disulfide bonds ensuing from the influence of metallic nanoparticles because the relatively flat surface of sGNP made contact with the disulfide bridge difficult in contrast to PoGNP’s rough surface.

### Antiviral effect of PoGNP towards various influenza A virus subtypes

**Fig. 5:**
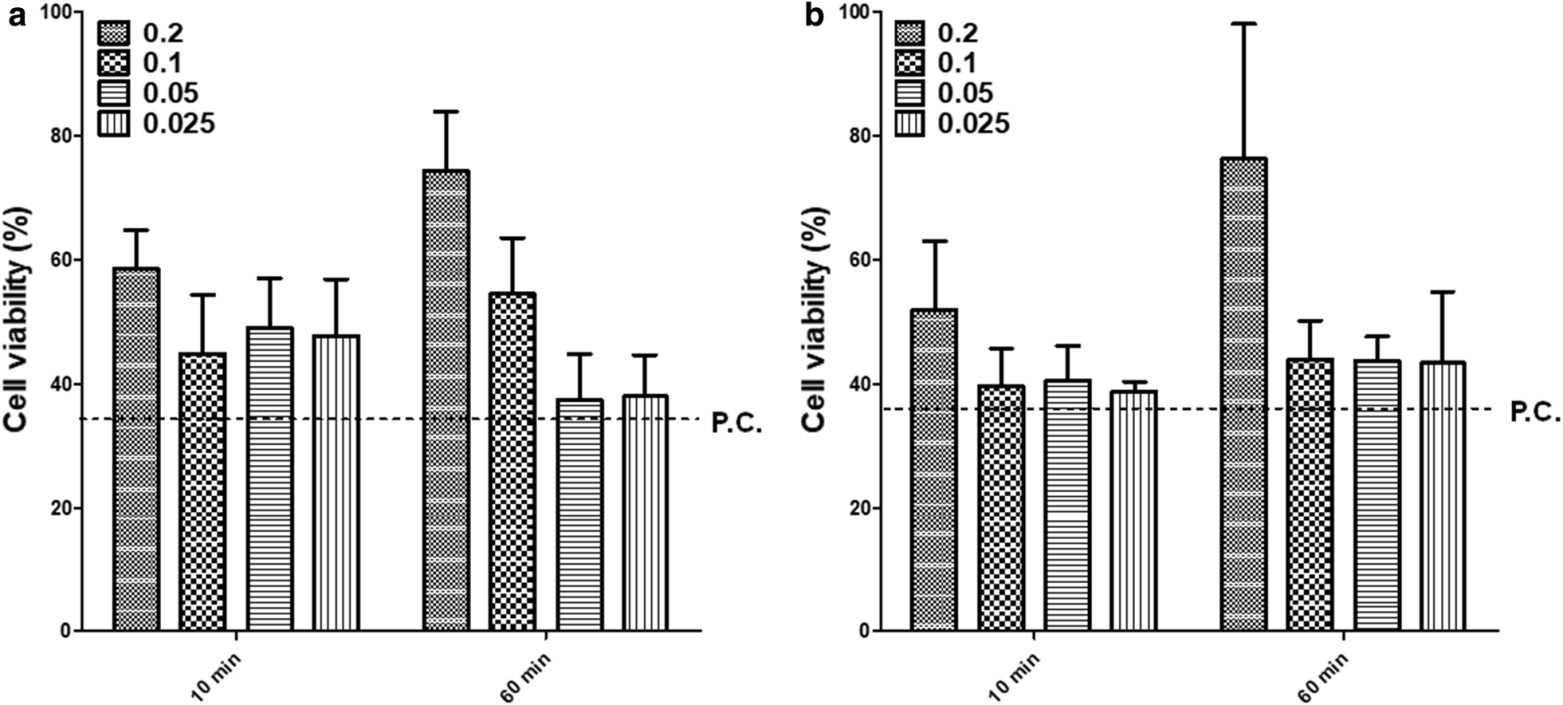
MDCK cell viability assay of PoGNP treatment for 10 min and 60 min on **a** H1N1, **b** H3N2 virus-infected cells. [Viral titer: 10^6^ EID_50_/mL, dotted line: positive control (P.C.)]

To confirm the inactivation of PoGNP regardless of genetic mutation, 3 IAV strains (H1N1, H3N2, and H9N2) were treated with PoGNP (Fig. 5 and Additional file 1: Figure S3). According to the phylogenic tree of IAV, the selected virus strains had low sequence similarity, so they were considered representative of general viral treatment [33]. For the 10 min-treated sample, only 0.2 mg/mL PoGNP showed over 50% cell viability. Under 0.1 mg/mL PoGNP concentration, the treated virus was only slightly inactivated compared to the non-treated virus. After the 60 min treatment, the antiviral effect of treatment with 0.2 mg/mL PoGNP on H1N1, H3N2, and H9N2 increased to 74%, 76%, and 56%, respectively. 54% of MDCK cells survived H1N1 infection when treated with 0.1 mg/mL PoGNP for 60 min. H3N2 and H9N2 were minorly affected under 0.1 mg/mL PoGNP and still exhibited higher cell viability than the control group. PoGNP displayed antiviral activity on various virus strains, which is important for on-the-spot preprocessing of IAV for further analysis. Here, we could conclude that 0.2 mg/mL PoGNP could attenuate the infectivity of multiple IAV strains.

### Intracellular viral RNA quantification

**Fig. 6:**
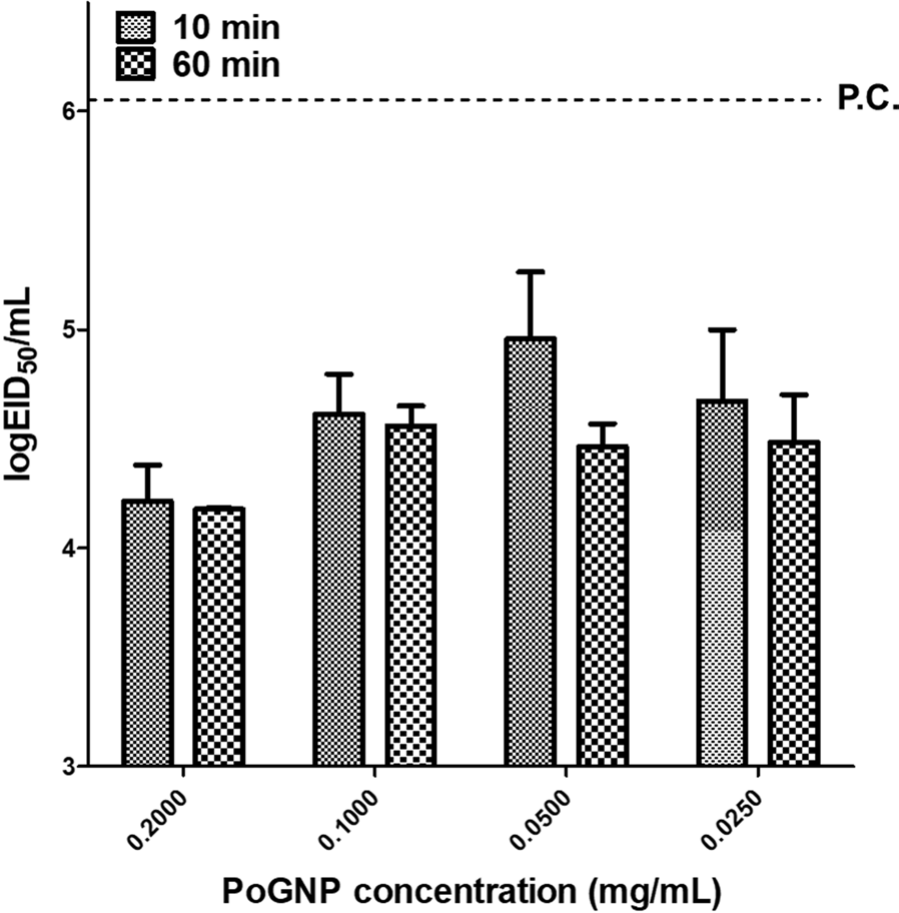
EID of intracellular viral RNA derived from real-time RT-PCR data, infected after PoGNP treatment. [Dotted line: positive control (P.C.)]. PoGNP, porous gold nanoparticle

We had inferred the inactivation mechanism of the virus as the cleavage of disulfide bonds, because the high affinity of the disulfide-gold interaction and abundant presence of disulfide bonds in HA would expedite inhibition by PoGNP. The viral inhibition mechanism of PoGNP was then identified by quantitative analysis of intracellular viral RNA from MDCK cells infected with nanoparticle-treated viruses (Fig. 6). MDCK cells were infected with PoGNP-treated H3N2 virus for 24 h and the cells were lysed with Qiagen RNeasy mini kit, followed by calculation of logEID_50_/mL values by standard EID curve (Additional file 1: Figure S4). PoGNP-treated H3N2 virus showed lower intracellular viral RNA levels indicating that the amount of IAV in the endosome of MDCK cells was clearly reduced in the nanoparticle-treated samples. Also, the amount of intracellular viral RNA was clearly influenced by the exposure time. The gene content level of viral RNA in the host cells was higher for viruses treated for 10 min compared with 60 min. Intracellular viral RNA quantification data corresponded well with the MDCK cell viability test results, revealing that the antiviral activity of PoGNP was affected by time and concentration, especially 0.2 mg/mL PoGNP was effective after exposure for 10 min. Furthermore, the result also confirmed that PoGNP blocked viral infection by inhibiting viral entry.

### Demonstrating the importance of disulfide bond in viral infection

**Fig. 7:**
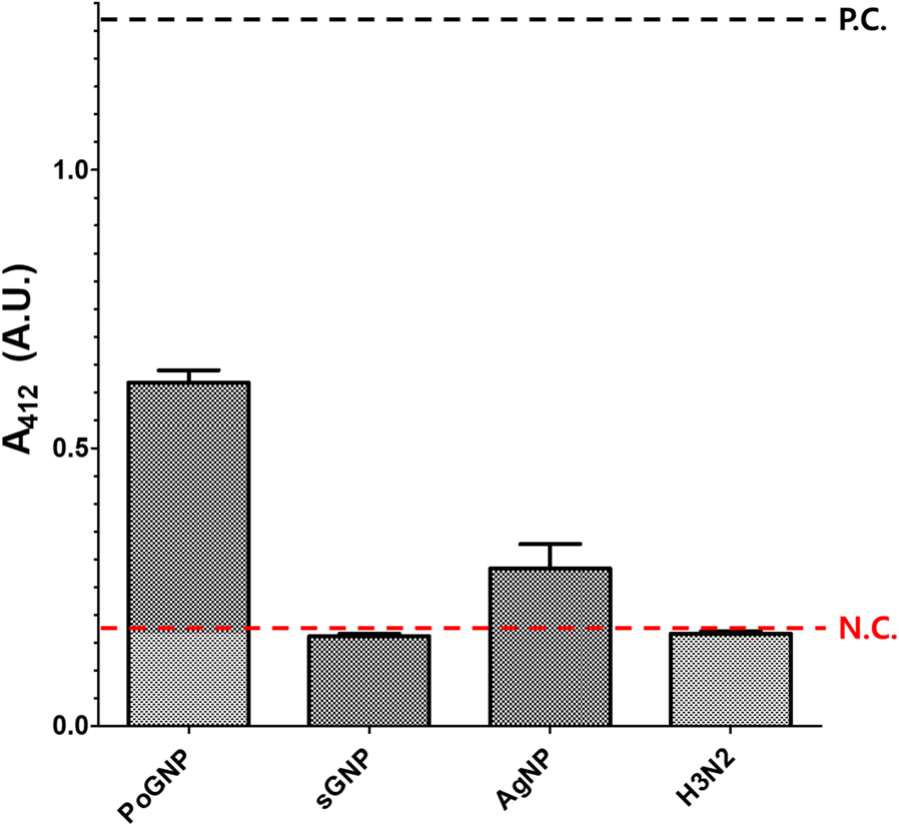
DTNB reduction assay performed with 10^6^ TCID_50_/mL of H3N2 that had reacted with 0.2 mg/mL of various nanoparticles for 60 min. (Black dotted line: positive control, 0.01 M TCEP with virus suspension; Red dotted line: negative control, 1 mM DTNB in 10% ethanol solution)

We determined that viral infection was suppressed by obstructing viral attachment, therefore we further experimented to prove that the disulfide bonds in HA have important role in viral infection. To support the hypothesis, 5,5′-dithiobis(2-nitrobenzoic acid) (DTNB) reduction assay (Ellman’s assay) was proposed to quantify the reduced disulfide bonds in virus hemagglutinin which had been in contact with 0.2 mg/mL of PoGNP, sGNP and AgNP (Fig. 7). As the higher sulfhydryl group concentration in the virus means the more reduction of disulfide bond has occurred, Ellman’s assay was considered as a valid method for proving relationship between viral disulfide bond reduction and its attenuated infectivity. As described in Fig. 7, absorbance at 412 nm (A_412_) of influenza virus that was exposed to 0.2 mg/mL PoGNP showed higher A_412_ than sGNP and AgNP counterpart at the same concentration, indicating that PoGNP have higher affinity to the disulfide bonds in HA. Despite the size of PoGNP slightly larger than sGNP, PoGNP’s higher relative surface area made a big difference in disulfide bond reduction while AgNP still displayed reductive reaction on the bonds in HA. TCEP was chosen as a positive control group of disulfide bond reducing agent since TCEP structure do not have any disulfide bond after reduced the S–S bond in HA, and thus it has little effect on DTNB reduction after reacting with disulfide bonds in HA.

Moreover, results of the WST-1 assay revealed that TCEP-treated IAVs lost their infectivity similar to PoGNP-treated viruses. Samples treated with under 1 mM TCEP exhibited nearly 60% cell viability, which was much higher than the 33% viability of H3N2 virus-infected MDCK cells (Additional file 1: Figure S5). On the other hand, 0.01 M TCEP-treated samples displayed lower cell viability compared to other TCEP-treated samples because of the cytotoxicity of TCEP itself [34]. The result suggested that the cleavage of disulfide bond before membrane fusion reduce viral infectivity toward MDCK cells. Likewise, as the viral infectivity of PoGNP-reacted influenza virus was suppressed to the level similar to that of the TCEP interacted one, which derives that PoGNP also cleaves the disulfide bonds in HA as TCEP.

## Conclusion

We demonstrated the antiviral activity of PoGNP in comparison with non-porous gold and silver nanoparticles in this study. Nanoparticle morphology and interaction with IAV was observed by TEM analysis and the interaction between PoGNP and HA was further proven by coprecipitation during centrifugation. Precipitation of PoGNP-treated IAVs showed lower real-time Cq values, indicating that more viruses precipitated with nanoparticles than were free in the supernatant. Furthermore, PoGNP showed more effective inhibition of viral infection compared with the non-porous sGNP and AgNP because of its extensive surface area. We suggested that the viral inhibition mechanism was due to blocking of viral attachment associated with membrane fusion, which ensued from the cleavage of disulfide bonds in HA. The results indicate that the disulfide bonds in HA could be an effective antiviral target, presenting possible development of antiviral vaccines for various IV strains. Likewise, we suggest that PoGNP could be applied to other enveloped viruses that have considerable spike proteins on the surface, such as human immunodeficiency virus or coronavirus, by cleaving disulfide bonds in their spike proteins. In addition, PoGNP has numerous electromagnetic hot spots compared to non-porous gold and silver nanoparticles, and the application of surface enhanced Raman spectroscopy (SERS) should be considered for future analysis. The findings of this study imply that a facile detection of IAV can be achieved by developing PoGNP as a multiplatform for detection and inactivation of the virus.

## Methods

### Materials

Gold (III) chloride trihydrate, silver (I) nitrate, tannic acid, sodium citrate, hydroquinone, polyvinylpyrrolidone (PVP), phosphotungstic acid hydrate (PTA), l-cysteine, monosodium phosphate, disodium phosphate and aniline were purchased from Sigma-Aldrich (St. Louis, USA). Ethanol and *N*-methyl-2-pyrrolidone (NMP) was obtained from Duksan Pure Chemicals Co., Ltd. (Ansan, South Korea). Dulbecco’s Modified Eagle Medium (DMEM), Dulbecco’s phosphate buffered saline (DPBS, pH 7.4), and fetal bovine serum (FBS) were purchased from Gibco Laboratories (Gaithersburg, USA). Ellman’s reagent [5,5′-dithiobis(2-nitrobenzoic acid), DTNB] was purchased from Thermo Scientific (Waltham, USA). The Ez-cytox cell viability assay kit was purchased from Daeil Lab Service (Seoul, Korea). The RNeasy^®^ mini kit and the QIAamp^®^ viral RNA mini kit were purchased from QIAGEN (Hilden, Germany). A/California/04/2009 (H1N1), A/canine/Korea/GCVP01/2007(H3N2) and A/wild bird feces/Korea/KU-VI135874/2012(H9N2) were prepared.

### Synthesis of metallic nanoparticle

PoGNP was prepared following the surfactant-free emulsion method [29]. Aniline monomer (0.182 mL, 2 mmol) was dispersed in 20 mL of deionized water. Then, 1 mL of gold (III) chloride solution (0.1 M) was added to construct a polyaniline (PANI)-Au hybrid nanoparticle under emulsification for 30 min with ultrasonication. The produced PANI-Au nanoparticle was washed 3× by centrifugation. PANI was then removed from the PANI-Au composite by dissolving the composite in NMP. Finally, purified PoGNP was redispersed in DPBS.

AgNP was synthesized following the citrate reduction method [35]. Tannic acid (42 mg, 0.025 mmol) and sodium citrate (150 mg, 0.5 mmol) were dissolved in 100 mL of deionized water, and the solution was heated to 95 °C with vigorous stirring. Then 1 mL of silver (I) nitrate solution (25 mM) was quickly injected and reacted for 1 h at 95 °C.

Non-porous sGNP was obtained following the seed-mediated growth method [36]. First, gold nanoparticle (GNP) seed was synthesized using the citrate reduction method. Briefly, 100 mL of sodium citrate solution (5 mM) was heated to 95 °C with vigorous stirring. Thereafter, 1 mL of gold (III) chloride solution (0.1 M) was injected into the sodium citrate solution. After 30 min, a wine-colored GNP seed solution was obtained. Then, 100 μL of gold (III) chloride solution (25 mM) was added to GNP seed solution (0.8 μM) to give a final volume of 10 mL. Subsequently, 22 μL of sodium citrate solution (30 mM) was injected into the seed solution mixture, following the addition of 100 μL of hydroquinone solution (30 mM). The mixture was kept at room temperature (RT) for 24 h. PVP solution (2 μg/mL) was added to prevent the aggregation of nanoparticles, followed by centrifugation at 10,000 rpm for 10 min.

Size distribution and zeta potential of each nanoparticle was collected by ELS-Z2000 (Otsuka electronics Co., Ltd., Osaka), and concentration of particles were analyzed through an inductively-coupled plasma optical emission spectrometer (ICP-OES, Perkin Elmer, Waltham).

### Binding efficiency between nanoparticle and influenza virus

300 μL aliquots of H3N2 virus suspension were exposed to 2 × 10^−1^ mg/mL to 6.25 × 10^−3^ mg/mL of nanoparticle (PoGNP and sGNP) solution and incubated at RT for 1 h. Subsequently, the incubated samples were centrifuged at 8000 rpm for 10 min to separate the virus-bound nanoparticles from the supernatant solutions. The supernatants were transferred to other test tubes while the precipitates were dispersed in DPBS. Thereafter, real-time RT-PCR analysis was conducted on the prepared solutions to determine the binding efficiency of the nanoparticles.

### Cytotoxicity assay for antiviral activities

The water-soluble tetrazolium salt-1 (WST-1) assay was performed to determine the cytotoxicity of the nanoparticle-treated IAVs on MDCK cells using the Ez-cytox cell viability assay kit (Daeil Lab Service). Twenty-four hours prior to the infection experiment, 2.0 × 10^5^ of Madin-Darby canine kidney (MDCK) cells were seeded into 96-well plates with 100 μL of 10% fetal bovine serum (FBS) containing DMEM.

In order to evaluate the antiviral activity of the nanoparticles, various nanoparticle concentrations (3.125 × 10^−2^ mg/mL to 2 × 10^−1^ mg/mL of PoGNP, sGNP, and AgNP) with twofold serial dilutions and 300 μL aliquots of IAVs (H1N1, H3N2, and H9N2) were prepared. 75 μL of each nanoparticle solution was added to the virus aliquots, for a final nanoparticle concentration of 6.25 × 10^−3^ mg/mL to 2 × 10^−1^ mg/mL. Samples were incubated at RT for 10 min and 60 min, with vortex mixing every 10 min. In the meantime, the MDCK cells were washed 3× with 100 μL of PBS. Then 100 μL aliquots of nanoparticle-treated IAV samples were added to each well and incubated for an additional 1 h. The nanoparticle-treated IAVs were then removed, cells were rinsed once with PBS, and then incubated for an additional 72 h with 200 μL of fresh DMEM.

To determine cell viability, 20 μL of Ez-cytox was added to the incubated cells and their optical density (OD) at 450 nm was measured after 2 h using the UV–Vis SpectraMax 190 Microplate Reader (Molecular Devices, San Jose, USA).

For intracellular viral RNA quantification, a 24 h MDCK cell incubation period was followed by the RNeasy^®^ mini kit protocol (QIAGEN) to prepare samples for real-time RT-PCR analysis.

### Transmission electron microscopy (TEM) analysis

30 μL of the nanoparticle and virus suspension mixtures, as previously described in cytotoxicity assay part, were incubated at RT for 10 and 60 min and then transferred by pipette to carbon-coated copper TEM grids (400 mesh). After 10 min at RT, the liquid was blotted with filter paper and a droplet of 3 wt% PTA solution was loaded onto the grid for negative staining. The excess PTA solution was blotted with filter paper after 10–40 s, followed by washing 2× with a droplet of deionized water, and the grid was dried for 4 h prior to analysis. TEM images were obtained with a JEM-1011 transmission electron microscope (JEOL Ltd., Tokyo, Japan).

### Real-time RT-PCR

A/canine/Korea/GCVP01/2007(H3N2) virus was serially diluted tenfold (108.25–102.25 EID_50_/mL). Viral genomic RNA from each dilution was extracted using the QIAamp^®^ viral RNA mini kit (QIAGEN), according to the manufacturer’s instructions. Real-time reverse transcription-polymerase chain reaction (RT-PCR) was employed to quantify the viral load in the samples using the QuantiTect Probe RT-PCR Kit (QIAGEN) and the LightCycler 96 system (Roche, Basel, Switzerland). The 50 μL final reaction volume contained 0.4 μM of matrix (M) gene-specific primer (forward: GACCRATCCTGTCACCTCTGAC; reverse: AGGGC ATTYTGGACAAAKC GTCTA) and 0.2 μM of specific probe (FAM-TGCAGTCCTCGCTCACTGGGCACG-BHQ-1). The thermal cycling conditions were: reverse transcription at 50 °C for 30 min, initial denaturation at 95 °C for 5 min, followed by 40 cycles of [94 °C for 15 s, 60 °C for 60 s] according to the manufacturer’s protocol (Roche). The primers targeted the region of the M gene highly conserved across all IAV strains, as formulated by the CDC (Biosearch Technologies, Inc. http://www.who.int/csr/resources/publications/swineflu/CDCrealtimeRTPCRprotocol_20090428.pdf). A standard curve was generated with EID values and copy numbers. The amount of viral RNA in nanoparticle-treated samples (in logEID_50_/mL) was calculated from the standard curve generated by real-time RT-PCR data using canine H3N2 virus (Additional file 1: Figure S4).

### Ellman’s assay

10 mM of DTNB in 10% ethanol solution and 0.1 M sodium phosphate buffer pH 7.4 was prepared. Then 50 μL of 0.2 mg/mL PoGNP, sGNP and AgNP aliquot was mixed with 50 μL of H3N2 virus and left for 60 min at RT. 100 μL of 1 mM DTNB solution was set as a negative control for the signal, and 100 μL of H3N2 virus suspension reacted with 10 mM TCEP for 60 min was prepared as a positive control. Subsequently, 20 μL of 10 mM DTNB solution was added to the mixture. Centrifugation of reactant was followed to remove nanoparticles in the mixture. Next, absorbance at 412 nm (A_412_) was observed using the UV–Vis SpectraMax 190 Microplate Reader (Molecular Devices, San Jose, USA).

## Supplementary information

**Additional file 1: Figure S1.** Size distribution plot of (A) PoGNP (B) sGNP (C) AgNP; Polydispersity index (PDI) of each nanoparticle was 0.058, 0.032 and 0.048, respectively. **Figure S2.** MDCK cell viability after treatment with (A) PoGNP (B) sGNP and (C) AgNP at various nanoparticle concentrations. **Figure S3.** MDCK cell viability assay of PoGNP treatment for 10 min and 60 min on H9N2 virus infected cell. (viral titer: 106 EID50/mL, dotted line: positive control (P.C.)). **Figure S4.** Standard curve of EID value derived by real-time RT-PCR. **Figure S5.** MDCK cell viability after infection with TECP-treated H3N2 virus. Upper line: negative control (N.C.), lower line: positive control (P.C.). (viral titer: 10^6^ EID_50_/mL).

## Data Availability

All data generated or analyzed during this study are included in this published article and its supplementary information files.
