# Supplementary material for: Porous gold nanoparticles for attenuating infectivity of influenza A virus
Source: J Nanobiotechnology. 2020 Mar 24;18:54. doi: 10.1186/s12951-020-00611-8 (PMC7092597; doi:10.1186/s12951-020-00611-8)
Supplement: Supplementary file 1 — Additional file 1: Figure S1. Size distribution plot of (A) PoGNP (B) sGNP (C) AgNP; Polydispersity index (PDI) of each nanoparticle was 0.058, 0.032 and 0.048, respectively. Figure S2. MDCK cell viability after treatment with (A) PoGNP (B) sGNP and (C) AgNP at various nanoparticle concentrations. Figure S3. MDCK cell viability assay of PoGNP treatment for 10 min and 60 min on H9N2 virus infected cell. (viral titer: 106 EID50/mL, dotted line: positive control (P.C.)). Figure S4. Standard curve of EID value derived by real-time RT-PCR. Figure S5. MDCK cell viability after infection with TECP-treated H3N2 virus. Upper line: negative control (N.C.), lower line: positive control (P.C.). (viral titer: 106 EID50/mL). [file 12951_2020_611_MOESM1_ESM.docx]

Porous gold nanoparticles for attenuating infectivity of influenza A virus

**Additional Figures**


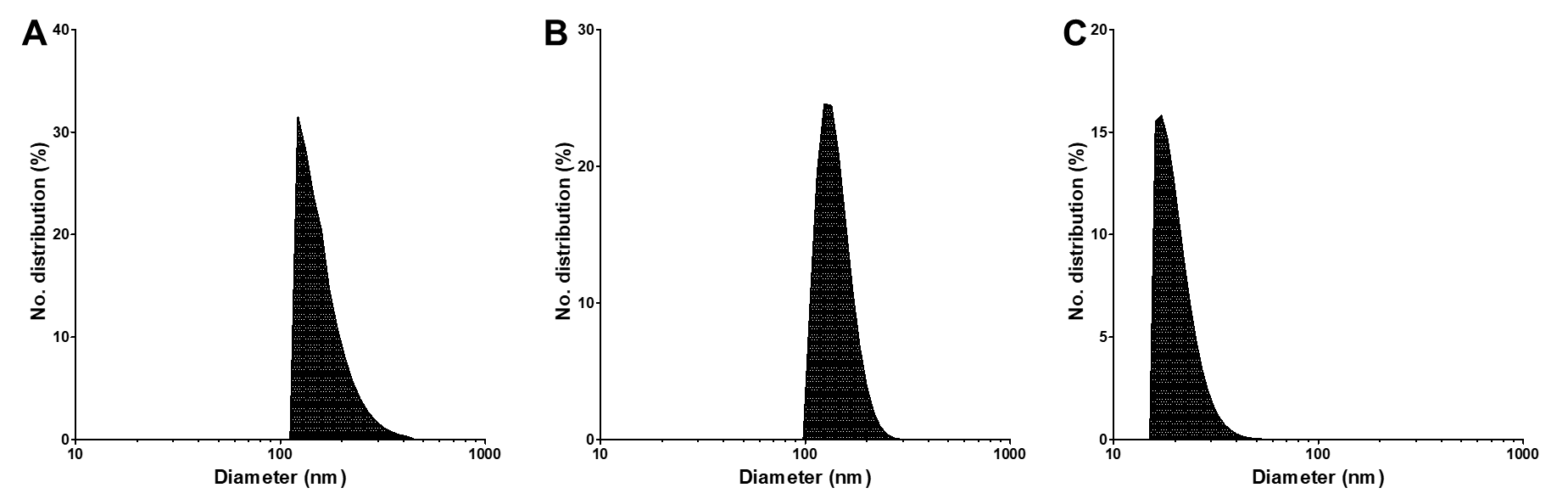


**Figure S1.** Size distribution plot of (A) PoGNP (B) sGNP (C) AgNP; Polydispersity index (PDI) of each nanoparticle was 0.058, 0.032 and 0.048, respectively.


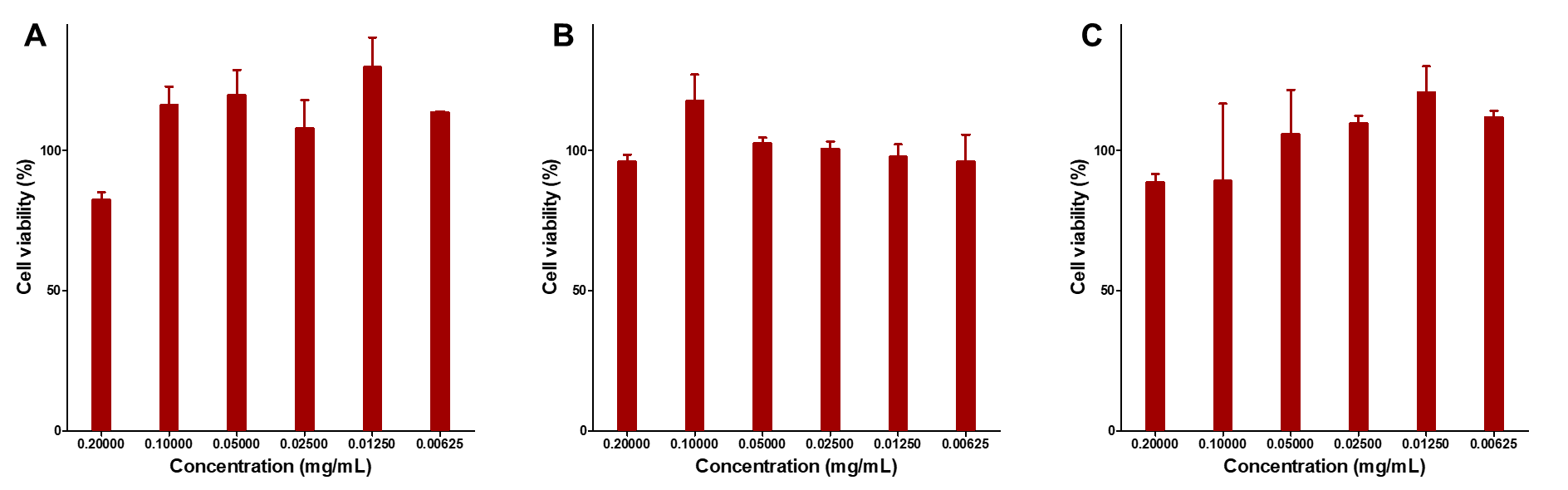


**Figure S2**. MDCK cell viability after treatment with (A) PoGNP (B) sGNP and (C) AgNP at various nanoparticle concentrations.


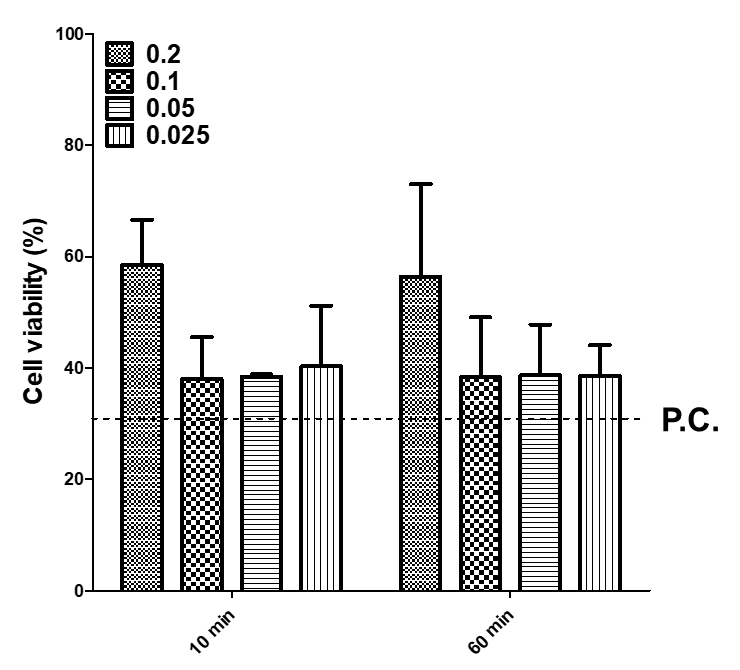


**Figure S3.** MDCK cell viability assay of PoGNP treatment for 10 min and 60 min on H9N2 virus infected cell. (viral titer: 10^6^ EID_50_/mL, dotted line: positive control (P.C.))


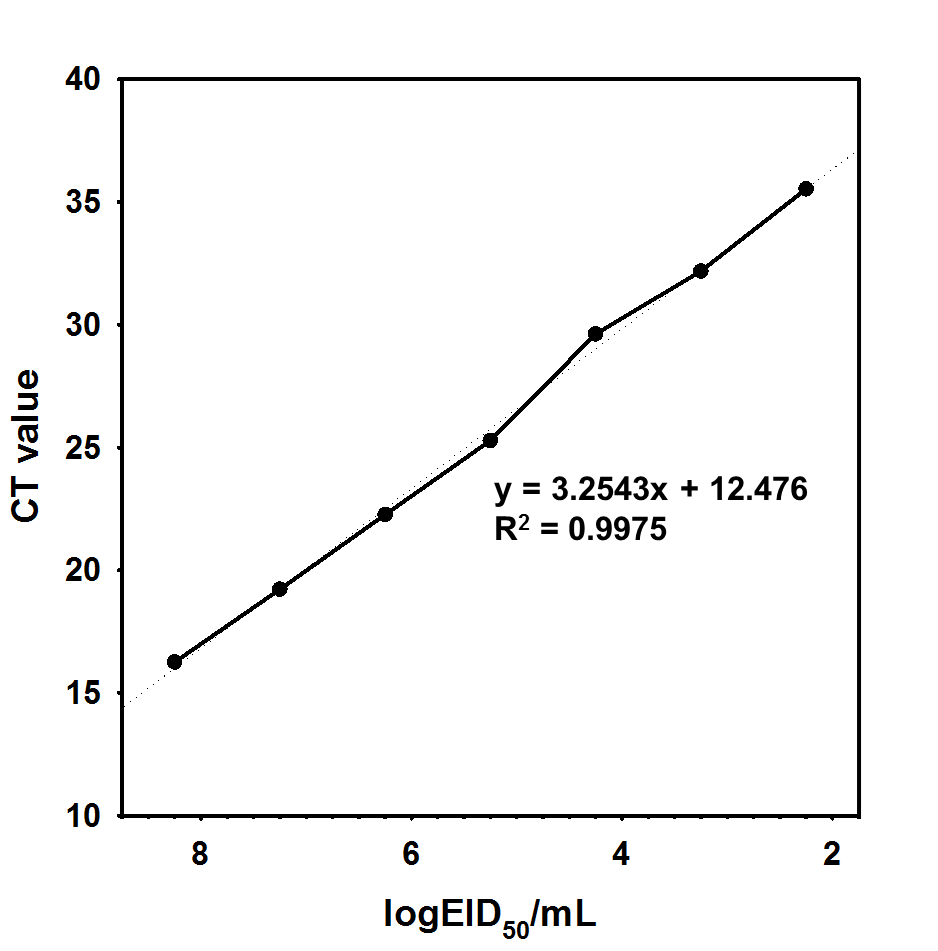


**Figure S4**. Standard curve of EID value derived by real-time RT-PCR.


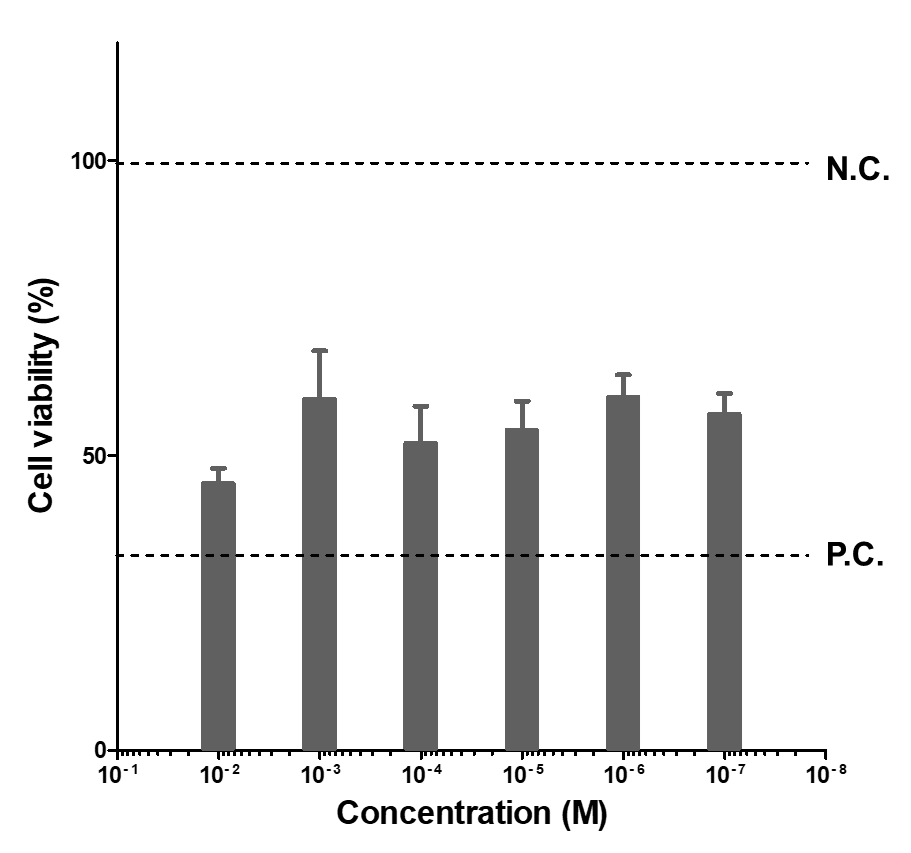


**Figure S5.** MDCK cell viability after infection with TECP-treated H3N2 virus. Upper line: negative control (N.C.), lower line: positive control (P.C.). (viral titer: 10^6^ EID_50_/mL)
